# Genetic susceptibility of Henoch-Schönlein purpura in children

**DOI:** 10.1186/1755-8166-7-S1-P65

**Published:** 2014-01-21

**Authors:** Ritu Aggarwal, Anju Gupta, Jasmine Naru, Neha Nanda, Manila Salaria, Deepti Suri, Surjit Singh

**Affiliations:** 1 Department of Immunopathology and Allergy and Immunology Unit, Deptartment of Pediatrics, Post Graduate Institute of Medical Education and Research, Chandigarh, India

## Background

Henoch-Schönlein purpura (HSP) is a small vessel vasculitis typically observed in children, 3-10 years old. The aetiology is unclear. Interaction of several environmental factors, including infections and multiple genes has been proposed to play a role in pathogenesis. An increased familial occurrence is an indicator of genetic predisposition; association with a major histocompatibility complex is plausible. The aim of the study was to investigate the association of HLA-DRB1 (HLA class II antigen) with HSP.

## Subjects and methods

The study was prospective and hospital based. Patients up to the age of 14 years, who fulfilled the diagnostic criteria of HSP, laid by the ‘European League Against Rheumatism’ were enrolled. Age matched healthy controls were included as well. One ml blood in EDTA was collected from patients as well as controls. DNA extraction was performed using commercially available kit. The quantity and quality of DNA was estimated by spectrophotometer and PCR for housekeeping gene beta actin, respectively. PCR with 24 sequence specific primers for HLA-DRB1 antigen was performed. Commercially available HLA-DR tissue typing kit (Inno-train, Kronberg im Taunus, Hesse, Germany) was utilized. Frequency of HLA-DRB1 was correlated with gastrointestinal and renal involvement.

## Results

The study included 43 patients and 53 controls. The mean age of the patients and controls was 8.5 years (range: 3-14) and 7.4 years (range: 1-14), respectively. Frequency of HLA-DRB1*11 was significantly increased in patients (p=0.006). A greater gastrointestinal (p=0.03) as well as renal (p=0.004) involvement was observed in patients with HLA-DRB1*11.

## Conclusion

This is the first study from India to report the HLA susceptibility genes in children with HSP. Presence of HLA-DRB1*11 was observed to predispose to HSP in children, as well as for greater likelihood of gastrointestinal and renal involvement.

